# Report from the Alzheimer’s Research UK Conference 2015

**DOI:** 10.1186/s13195-015-0138-x

**Published:** 2015-07-28

**Authors:** Rosa M. Sancho, Carla J. Cox, Simon H. Ridley, Laura E. Phipps, Eric Karran

**Affiliations:** Alzheimer’s Research UK, 3 Riverside, Granta Park, Cambridge, CB21 6 AD, UK

## Abstract

On 10–11 March 2015 University College London hosted the annual Alzheimer’s Research UK Conference. This report provides an overview of the presentations and discussions that took place.

## Introduction

The 2015 Alzheimer’s Research UK (ARUK) Conference offered a diverse programme, prepared by a committee led by Sebastian Crutch (University College London (UCL), UK). The programme started with the perspectives of four clinicians, who exposed the singularities and challenges presented by different causes of dementia. These were followed by the latest insights from genetics, pathology and brain imaging.

## Vascular pathology

Hugh Markus (University of Cambridge, UK) showed that patients with small vessel disease, in contrast to patients with Alzheimer’s disease (AD), present a neuropsychological profile with a more prominent deficit of executive function than episodic memory. Frederik Barkhof (VU University Medical Centre, the Netherlands) described neuroimaging features of patients with vascular dementia and discussed the interplay between vascular and AD pathologies, presenting a correlation between atherosclerotic calcification, cognition and structural brain changes.

From work carried out in collaboration with Southampton researchers, John Hardy (UCL, UK) suggested that seeding of amyloid-beta (Aβ) plaques and propagation of tau could be triggered by loss of homoeostasis due to age-related failure of lymphatic drainage along walls of aged cerebral arteries. James Nicoll (University of Southampton, UK) indicated that, in cerebral amyloid angiopathy (CAA), Aβ accumulation in blood vessel walls disrupted smooth muscle cells, which normally regulate local blood flow to meet neuronal metabolic demand. Nicoll also described seminal work on the analysis of neuropathology cases following AN1792 active immunisation. Whilst amyloid clearance was achieved, increased CAA and CAA-associated vasculopathy and amyloid-related imaging abnormalities (ARIA) suggested that plaques were removed from the brain by vascular routes. A reduction of plaque-associated apolipoprotein E (ApoE) and an increase of vascular ApoE in vessels were also shown. Nick Fox (UCL, UK) showed an IgG-titre dose–response whole brain tissue loss following the AN1792 and bapineuzamab trials, including ventricular enlargement. However, Fox argued that, in the bapineuzamab passive immunisation trial, these volume changes were driven by ARIA.

Karen Horsburgh (University of Edinburgh, UK) described work in mice with cilostazol, a phosphodiesterase inhibitor with beneficial effects on vascular health. Cilostazol improved spatial working memory and white matter function in mice, and dampened microglial proliferation in the corpus callosum after hypoperfusion.

## Frontotemporal dementia

Building on recent genetic discoveries, Julie Snowden (University of Manchester, UK) showed the relationship between the diverse clinical characteristics and the underlying pathologies of frontotemporal dementia (FTD) (tau, TDP-43 and FUS) and associated genetic mutations (*MAPT*, *C9orf72* and *GRN*). Drawing from neuropathology studies, Manuela Neumann (University of Tubingen, Germany) discussed findings in C9orf72 mutation cases suggesting a close correlation between the anatomical distribution of TDP-43 with the pattern of neurodegeneration and clinical phenotype. In contrast, the distribution of C9orf72 di-peptide repeats and RNA foci was highly consistent among cases, but showed no clinical correlation (age at onset or duration) or association with neurodegeneration. Neumann also suggested a key role for TDP-43 dysfunction as a downstream effect in C9orf72 mutation-associated pathogenesis. Using a newly generated Drosophila model, Alan Stepto (Frank Hirth’s laboratory, King’s College London (KCL), UK) reported that pan-neuronal expression of 64-repeat and 128-repeat, but not eight-repeat, hexanucleotide expansions of C9orf72 resulted in age-related behavioural deficits that correlated with repeat-associated non-ATG translation of di-peptide repeats. Additionally, Stepto observed premature death and impaired locomotor behaviour in flies expressing high levels of 38-repeat RNA. Chris Shaw (KCL, UK) revealed that seven missense mutations have been identified in an undisclosed calcium binding protein in familial and sporadic cases of motor neurone disease. Pathological analysis of one case indicated that the protein formed fibrillary and globular inclusions in neurones and glia as well as classical TDP-43 pathology.

## Dementia with Lewy bodies and synucleinopathies

John O’Brien (University of Cambridge, UK) highlighted the ongoing DIAMOND-Lewy programme (Improving the DIAgnosis and Management Of Neurodegenerative Dementia of Lewy body type, funded by the National Institute for Health Research) which aims to bring together an integrated diagnostic toolkit and a patient management pathway. As part of this study, which is a collaboration with Newcastle University and Durham University, O’Brien described an ongoing systematic review of dementia with Lewy bodies/Parkinson’s disease dementia trials, in which controlled trials show that cholinesterase inhibitors may have beneficial cognitive effects while uncontrolled trials suggest that levodopa could be helpful for motor symptoms. As a potential diagnostic tool, David Klenerman (University of Cambridge, UK) showed that, despite the presence of β-sheet oligomers in control samples, total β-sheet α-synuclein oligomers increased in size and number in Parkinson’s disease cerebrospinal fluid (CSF) samples.

## Alzheimer’s disease and atypical variants

Illustrated by cases from his own practice, Chris Butler (University of Oxford, UK) discussed the typical but clinically heterogeneous features of AD and the tools that can be used to detect them. Nick Fox (UCL) showed how imaging techniques are accurate in revealing hippocampal atrophy up to 5 years before the onset of symptoms with an annual reduction of 3–4 % as opposed to 0.3 % in healthy people. Imaging in posterior cortical atrophy showed widespread cortical amyloid deposition but more selective tau distribution, suggesting that tau is more closely linked to hypometabolism and symptomatology. Henrik Zetterberg (University of Gothenburg, Sweden and UCL, UK) showed that, in AD, CSF Aβ42 levels correlated with the presence of amyloid positron emission tomography and with postmortem amyloid cortical plaque load. Zetterberg also presented the C-terminus of the dendritic protein neurogranin as a potential biomarker for synaptic degeneration across neurodegenerative diseases and showed particular correlation with cognitive decline. The 2015 Nobel laureate John O’Keefe (UCL, UK) described his seminal work on spatial cells in hippocampal formation, now being translated into AD diagnostic markers. O’Keefe described how the 4 Mountains Test, a topographical memory task, was highly sensitive to focal hippocampal pathology and had high diagnostic sensitivity and specificity in differentiating mild cognitive impairment with and without CSF biomarkers.

At a molecular level, James Duce (University of Leeds, UK) presented evidence that iron disruption in AD could be modulated by the processing of amyloid precursor protein (APP) on the cell surface whereby the amyloidogenic pathway that requires BACE1 and leads to Aβ generation also causes intraneuronal iron retention and increases vulnerability to reactive oxygen species neurotoxicity; a common concomitant in AD. Rob Andrew (Nigel Hooper’s laboratory, University of Manchester, UK) investigated the interactomes of the APP695 and APP751 isoforms to clarify the mechanisms underpinning their differential processing to Aβ. Despite a significant overlap, the role of APP695 in nuclear signalling and mitochondrial functions was consolidated and higher levels were reported at the plasma membrane than for APP751. Bart De Strooper (KU Leuven, Belgium and UCL, UK) described the identification of G-protein coupled receptor 3 (GPR3) as a mediator of Aβ generation through interaction with β-arrestin 2. Subsequent work showed that GPR3 modulation, unlike other γ-secretase modulators, did not impact on notch signalling. Human brain samples showed increased GPR3 expression, and evidence from five mouse models showed that lower GPR3 expression reduced plaque burden, soluble Aβ and behavioural deficits.

During the session on tau, Karen Duff (Columbia University, NY, USA) showed that rTgTauEC, a model with predominant expression of abnormal tau in the entorhinal cortex, has reduced place and grid cell firing and that firing patterns across days were less specific, coherent and stable compared with control aged mice. Using these mice crossed with APP/PS1 mice, Amy Pooler (Nestlé Institute of Health Sciences, Switzerland; previously KCL, UK) found that at 16 months concurrent amyloid deposition in the cortex led to an increase in the speed of tau propagation to distal areas, and an increase in tau-induced neuronal loss. Duff showed data suggesting that tau accumulated on spine-like structures on the axon and was taken up by cells after a lag phase. In-vitro data presented by Tara Spires-Jones (University of Edinburgh, UK) provided further evidence that tau was required for Aβ-induced synapse loss, possibly mediated by caspase 3. Spires-Jones also showed that the plaque size, dystrophic neurites per plaque and the amount of soluble Aβ in synaptoneurosomes were increased in APP/PS1/hTau mice compared with APP/PS1 mice, correlating with data presented by Pooler. It was suggested that amyloid accumulation could trigger neuronal activity and therefore release of tau. In the P301S mouse model, Maria Grazia Spillantini (University of Cambridge, UK) determined that several proteins were differentially expressed between 1 and 5 months, as tau pathology and neuronal death progressed. Spillantini also showed that astrocytes and astrocyte culture medium from these mice were toxic to neurones, with a reduction of synaptic protein function.

Dominic Walsh (Harvard University, MA, USA) kick-started the annual debate ‘Are oligomers the real target?’ arguing for the motion of Aβ oligomers as the toxic species. He suggested that Aβ, acting as an initiator of AD, would not have to show direct correlation with clinical severity. Walsh showed that certain APPTg mice exhibit cognitive deficits prior to appreciable amyloid deposition and benefit from acute treatment with anti-Aβ monoclonal antibodies, and that human brain-derived, non-fibrillar Aβ has been shown to have disease-relevant activity. De Strooper, arguing against the motion, disputed evidence of Aβ toxicity, citing the gap in concentrations and time courses observed in in-vitro models versus the human disease. He further argued that the large variety of receptors reported to bind Aβ and mediate its effects cannot be specific effects of Aβ. The lively debate highlighted important barriers to overcome, including lack of experimental standardisation and integration across laboratories as well as lack of basic knowledge of oligomer biochemistry and the relationship between Aβ and cognitive decline.

## Commonalities

Keynote speaker Hardy demonstrated how selective vulnerability may, in part, be a consequence of different neurons being close to differing ‘catastrophic cliffs’ dependent on their function. As an example, Hardy described work using transcriptomics across the human brain that identified a *RAB39B*-containing module in the substantia nigra enriched for genes implicated in mitochondrial function, including *SNCA*, *PARKIN* and *PINK1*. Rita Louro Guerreiro (UCL, UK) showed pathway and network analyses that identified immune response modules as the most significant in AD. Guerreiro also highlighted the finding that the neuroinflammation positron emission tomography ligand PK11195 was not effective in 30 % of a Caucasian population owing to a polymorphism in the translocator protein, suggesting that integration of genetic findings is essential to improve diagnosis. Tony Wyss-Coray (Stanford University, CA, USA) presented various analyses of human plasma samples from the umbilical cord and from adults aged 20 and 65 which indicated that prominent changes in secreted signalling proteins correlated with ageing, including an increase in inflammatory proteins and a decrease in growth factors. Wyss-Coray also showed that, in aged NOD SCID gamma mice subjected to heterochronic parabiosis, circulatory plasma factors from young humans could ameliorate cognitive deficits, increase hippocampal expression of plasticity-related genes and increase the number of c-fos-expressing neurons in the dentate gyrus. One of the ‘rejuvenation factors’ in young plasma was identified as colony-stimulating factor 2.

## Resources and initiatives

Eric Karran (ARUK) provided an update on ARUK’s initiatives and highlighted the launch of the Drug Discovery Alliance. Three Lead Academic Scientists of the Alliance—David Rubinsztein (University of Cambridge, UK), Giampietro Schiavo (UCL, UK) and Chas Bountra (University of Oxford, UK)—spoke of their plan to establish Drug Discovery Institutes at their respective institutions and to establish a network with academia and industry to facilitate the progression of lead molecules towards the clinic.

In a session dedicated to resources, Paul Francis (KCL, UK) presented Brains for Dementia Research (http://www.brainsfordementiaresearch.org.uk), Dervis Salih (UCL, UK) described Mouseac (www.Mouseac.org) and Braineac (www.Braineac.org), Cathie Sudlow (University of Edinburgh, UK) presented the UK Biobank (www.ukbiobank.ac.uk) and Jonathan Schott (UCL, UK) presented a range of imaging resources and image analysis software available at UCL (http://cmictig.cs.ucl.ac.uk/research/software).

## Abbreviations

AD: Alzheimer’s disease
ApoE: Apolipoprotein E
APP: Amyloid precursor protein
ARIA: Amyloid-related imaging abnormalities
ARUK: Alzheimer’s Research UK
Aβ: Amyloid-beta
CAA: Cerebral amyloid angiopathy
CSF: Cerebrospinal fluid
FTD: Frontotemporal dementia
GPR3: G-protein coupled receptor 3
KCL: King’s College London
UCL: University College London

